# The Influence of Monoamine Oxidase Variants on the Risk of Betel Quid-Associated Oral and Pharyngeal Cancer

**DOI:** 10.1155/2014/183548

**Published:** 2014-10-15

**Authors:** Ping-Ho Chen, Bin Huang, Tien-Yu Shieh, Yan-Hsiung Wang, Yuk-Kwan Chen, Ju-Hui Wu, Jhen-Hao Huang, Chun-Chia Chen, Ka-Wo Lee

**Affiliations:** ^1^School of Dentistry, College of Dental Medicine, Kaohsiung Medical University, No. 100 Shih-Chuan 1st Road, Kaohsiung 80708, Taiwan; ^2^Cancer Center, Kaohsiung Medical University Hospital, Kaohsiung Medical University, No. 100 Shih-Chuan 1st Road, Kaohsiung 80708, Taiwan; ^3^Global Center of Excellence for Oral Health Research and Development, No. 100 Shih-Chuan 1st Road, Kaohsiung 80708, Taiwan; ^4^Department of Biomedical Science and Environmental Biology, College of Life Science, Kaohsiung Medical University, No. 100 Shih-Chuan 1st Road, Kaohsiung 80708, Taiwan; ^5^Department of Biological Sciences, National Sun Yat-sen University, No. 70 Lienhai Road, Kaohsiung 80424, Taiwan; ^6^School of Oral Hygiene, Taipei Medical University, No. 250 Wuxing Street, Taipei 11031, Taiwan; ^7^Orthopaedic Research Center, College of Medicine, Kaohsiung Medical University, No. 100 Shih-Chuan 1st Road, Kaohsiung 80708, Taiwan; ^8^Division of Oral Pathology & Diagnosis, Department of Dentistry, Kaohsiung Medical University Hospital, No. 100 Shih-Chuan 1st Road, Kaohsiung 80708, Taiwan; ^9^Department of Oral Hygiene, College of Dental Medicine, Kaohsiung Medical University, No. 100 Shih-Chuan 1st Road, Kaohsiung 80708, Taiwan; ^10^Department of Dentistry, Kaohsiung Medical University Hospital, No. 100 Shih-Chuan 1st Road, Kaohsiung 80708, Taiwan; ^11^Institute of Biomedical Sciences, National Sun Yat-sen University, No. 70 Lienhai Road, Kaohsiung 80424, Taiwan; ^12^Division of Traumatology & Plastic Surgery, Department of Surgery, Chi Mei Medical Center, No. 901 Zhonghua Road, Yongkang District, Tainan 710, Taiwan; ^13^Institute of Imaging and Biomedical Photonics, College of Photonics, National Chiao Tung University, 1001 University Road, Hsinchu 30010, Taiwan; ^14^Department of Otolaryngology, Kaohsiung Medical University Hospital, No. 100 Shih-Chuan 1st Road, Kaohsiung 807, Taiwan; ^15^Department of Otolaryngology, College of Medicine, Kaohsiung Medical University, No. 100 Shih-Chuan 1st Road, Kaohsiung 80708, Taiwan

## Abstract

Betel quid (BQ) and areca nut (AN) (major BQ ingredient) are group I human carcinogens illustrated by International Agency for Research on Cancer and are closely associated with an elevated risk of oral potentially malignant disorders (OPMDs) and cancers of the oral cavity and pharynx. The primary alkaloid of AN, arecoline, can be metabolized *via* the monoamine oxidase (MAO) gene by inducing reactive oxygen species (ROS). The aim of this study was to investigate whether the variants of the susceptible candidate MAO genes are associated with OPMDs and oral and pharyngeal cancer. A significant trend of MAO-A mRNA expression was found in *in vitro* studies. Using paired human tissues, we confirmed the significantly decreased expression of MAO-A and MAO-B in cancerous tissues when compared with adjacent noncancerous tissues. Moreover, we determined that MAO-A single nucleotide polymorphism variants are significantly linked with oral and pharyngeal cancer patients in comparison to OPMDs patients [rs5953210 risk G-allele, odds ratio = 1.76; 95% confidence interval = 1.02-3.01]. In conclusion, we suggested that susceptible MAO family variants associated with oral and pharyngeal cancer may be implicated in the modulation of MAO gene activity associated with ROS.

## 1. Introduction

Oral and pharyngeal cancer is one of the most prevalent cancers in the world. In Taiwan, cancers of the oral cavity and pharynx were the fourth most prevalent cancers among males [1]. In 2010, the age-standardized incidence rate was estimated to be 40.56 per 100,000 persons (adjusted by the world population in 2000) for oral and pharyngeal cancer in Taiwanese males [1]. Also, the age-standardized mortality rate of males for oral and pharyngeal cancer in 2010 was 14.71 per 100,000, which leads to oral and pharyngeal cancer being ranked as the fourth leading cause of death due to cancer. Several studies suggested that betel quid (BQ) use may increase the risk of cancers of the oral cavity and pharynx and of oral potentially malignant disorders (OPMDs), including erythroplakia, leukoplakia, lichen planus, and oral submucous fibrosis (OSF) [2–4]. In addition, malignant transformation of OPMDs can result in the occurrence of oral and pharyngeal cancer [5].

There are approximately 600 million BQ chewers in the world [6]. Following nicotine, alcohol, and caffeine, BQ chewing is the fourth most frequently used addictive and psychoactive substance in the world [7]. BQ and AN (the major ingredients in various methods of BQ chewing) have been evaluated as group I carcinogens for humans by the International Agency for Research on Cancer [2]. In mammalian cells, arecoline was major alkaloid in AN, and it can induce cytotoxicity [8–10]. In human endothelial cells, the effects of cell cycle arrest, cytotoxicity, and apoptosis could be induced by arecoline treatment [11]. Arecoline is the major compound among the AN alkaloids, and it may be metabolized by MAO gene* via* xenobiotic metabolism, which is involved in phase I biotransformation [12]. AN extract or arecoline induces cell necrosis through increasing reactive oxygen species (ROS) [13] and ROS may be produced by MAO catalysis [14]. Microarray analysis screening data indicated that 100 *μ*g/mL arecoline treatment in a commercial normal human gingival fibroblast (HGF) cell line may induce MAO-A gene expression [12]. Therefore, we assume that the MAO-A gene may be associated with arecoline induction in oral cells and may be implicated in the occurrence or development of oral and pharyngeal cancer. To the best of our knowledge, no study has examined the correlation between the MAO gene variations and oral and pharyngeal cancer or OPMDs. The specific aim of this paper was to investigate whether susceptible MAO genes are associated with oral and pharyngeal cancer and OPMDs.

## 2. Materials and Methods

### 2.1. Study Subjects

This study was approved by the Ethical Review Committee of the Institutional Review Board (IRB) of Kaohsiung Medical University (KMU) Chung-Ho Memorial Hospital (KMUH-IRB-950094, KMUH-IRB-950315, and KMUH-IRB-970413). A total of 260 male patients diagnosed with oral and pharyngeal cancer and 68 male patients diagnosed with OPMDs participated in the study. Males with oral and pharyngeal cancer or OPMDs were selected from the Department of Otolaryngology and Division of Oral and Maxillofacial Surgery, Department of Dentistry at KMU Hospital. In this study, all participants agreed to undersign a written informed consent. All cases of oral cancer or OPMDs were histologically confirmed by pathologists or surgeons. By signing the informed consent, all subjects agreed to answer a questionnaire administered by trained interviewers and to provide blood samples for experimental analysis. Additionally, the informed consent permitted the collection of oral cancerous tissue and noncancerous adjacent oral tissue (a safe margin) from cancer patients during necessary surgery resection. Oral cancerous tissue and adjacent noncancerous tissues were collected without radiation therapy or chemotherapy.

### 2.2. Isolation and Culture of Human Gingival Fibroblasts (HGF)

Normal gingival tissue samples were obtained from the biopsy specimens during periodontal surgery on healthy subjects, all of whom provided informed consent. The study was approved by the hospital ethics committee (KMUH-IRB-20110031). HGF were isolated following a previously described method with some modifications [15, 16].

### 2.3. Cytotoxicity Assay

Normal clinical oral tissue was isolated to culture human gingival fibroblasts (HGF). Oral epidermal gingival squamous carcinoma, Ca9-22 cell line, was purchased from the Cell Bank of Japanese Collection of Research Bioresources (JCRB number JCRB0625). The details of our cell culture method were shown in our previous study [17]. Cells were treated with various concentrations (0, 50, 100, 200, 400, and 800 *μ*M) of arecoline incubated for 24 h and 48 h. The MTT was used to evaluate the cell proliferation for 2 h in CO_2_ incubator (37°C). In an ELISA reader (Bio Tek el800), cells were treated by using DMSO, and the absorbance (570 nm) was explored with the wavelength of reference (630 nm) after the removal of the culture medium. The percent of viable cells was shown in comparison with the vehicle controls.

### 2.4. Real-Time qRT-PCR Analysis

Following the commercial protocol of the manufacturer, the total RNA was extracted from the cells and tissues using TRIzol (Invitrogen, Carlsbad, CA, USA) as described [18]. Each cDNA pool was stored at −20°C until further qRT-PCR analysis. The primer pairs of specific oligonucleotide were selected from Roche Universal ProbeLibrary for qRT-PCR assays. The reactions of qRT-PCR using SYBR Green I kit were performed on the Roche LightCycler Instrument 1.5 system. The expression or repression of the target gene compared to the GAPDH gene (internal control) was calculated by the formula: 2^−ΔΔ*C*_*p*_^, where Δ*C*_*p*_ = *C*_*p*__target  gene_ − *C*_*p*__internal  control_ and ΔΔ*C*_*p*_ = Δ*C*_*p*__cancerous  tissue_ − Δ*C*_*p*__adjacent  noncancerous  tissue_ in each sample.

### 2.5. Protein Extraction and Western Blotting

The cell samples were washed with wash buffer [10 mM HEPES, pH 7.4, containing 140 mM NaCl, 4 mM KCl, and 11 mM glucose]. Cell lysate samples were obtained by sonication in lysis buffer [250 mM HEPES, pH 7.7, containing 1 mM EDTA, 0.1 mM neocuproine, and 0.4% (w/v) CHAPS]. The protein concentration was determined using the BCA protein assay reagent (Thermo Fisher Scientific Inc., Rockford, IL, USA). The cell lysate (40 *μ*g) was mixed with SDS-PAGE sample buffer [62.5 mM Tris-HCl, pH 6.8, 3% (w/v) SDS, 5% (v/v) 2-mercaptoethanol, and 10% (v/v) glycerol] and was then separated by SDS-PAGE. The blotted membranes were hybridized with monoclonal antibodies (Merck Millipore Corporation, Billerica, MA, USA), developed with the SuperSignal West Femto reagent (Thermo Fisher Scientific Inc.), and exposed to X-ray films. The images on X-ray films from three replicates were scanned using a digital scanner (Microtek International Inc., Hsinchu, Taiwan) and were analyzed using Progenesis Samespots v2.0 software (NonLinear Dynamics) to determine the level of protein expression.

### 2.6. Statistical Analysis

The assay results of MAO gene expression were presented as the mean and standard errors (SE) of the mean (mean ± SE) for each group. Between the control and treatment groups, one-way ANOVA and Tukey's honestly significant difference (HSD) test for post hoc multiple comparisons were used to evaluate statistical significance of the relative fold change. We also estimated the trend of dose-dependent effects for cell viability and MAO-A mRNA expression using a Cochran-Armitage trend test (*P* for trend). Because of the small sample size (*N* = 8) for the paired tissue, we conducted a nonparametric Wilcoxon signed-rank test to compare the protein expression differences between cancer tissue and its adjacent tissue.

The association between allele and diseases was estimated by chi-square (*χ* ^2^) test and an unconditional logistic regression model; odds ratio (OR), 95% confidence interval (CI), and exact *P* value were estimated. All statistical analysis was carried out using the IBM SPSS Statistics 19 (SPSS, Chicago, IL) and SAS Statistical Package (Version 9.1.3, SAS Institute Inc., Cary, NC, USA). Results that were considered significantly statistically different were marked with an asterisk (*P* < 0.05).

## 3. Results

### 3.1. HGF and Ca9-22 Cells Viability

**Figure 1:**
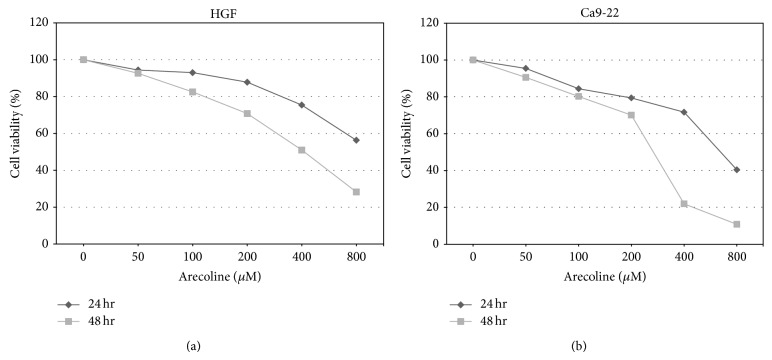
The effects of different arecoline concentrations on the viability of HGF and Ca9-22 cells for 24 h and 48 h. (a) HGF cells viability. (b) Ca9-22 cells viability. An asterisk (∗) indicates a statistically significant difference (*P* < 0.05).

MTT assay was used to estimate cell viability (%) after HGF and Ca9-22 cells exposure to six different concentrations (0, 50, 100, 200, 400, and 800 *μ*M) of arecoline for 24 h and 48 h. After the arecoline concentration was increased, cell survival gradually decreased in a time- and dose-dependent manner (Figure 1).

### 3.2. The mRNA Expression of MAO-A in HGF Cells and Oral Cancer Cell Lines (Ca9-22)

**Figure 2:**
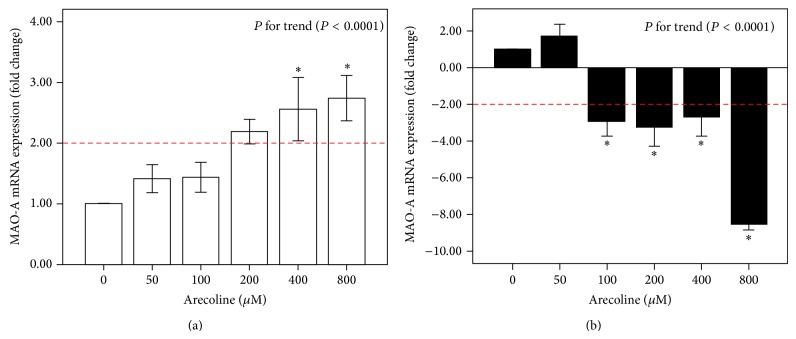
The mRNA expression of MAO-A after arecoline treatment at different concentrations (0, 50, 100, 200, 400, and 800 *μ*M). (a) Normal human gingival fibroblast cells (HGF). (b) Cancer cells (Ca9-22 cell line). The average fold change (mean ± standard errors (SE)) of the MAO-A gene was measured in triplicate; error bars indicate SE of mean. Multiple comparisons of mean MAO-A expression were analyzed by one-way ANOVA, and a post hoc comparison was performed by Tukey's HSD test. The *P* value for the trend is presented, and an asterisk (∗) indicates a statistically significant difference (*P* < 0.05) compared with cells without treatment.

Figure 2 showed that, at 200, 400, and 800 *μ*M arecoline, the expression of MAO-A was above 2-fold and was statistically significant at the 400 and 800 *μ*M doses in HGF cells (*P* < 0.05). An increasing trend effect (*P* < 0.0001) for MAO-A expression could be observed in HGF cells when the arecoline dose increased gradually. In cancer cell lines (Ca9-22), compared with the untreated control group, mRNA expression of MAO-A was increased slightly at 50 *μ*M. Conversely, a greater than 2-fold change in the downregulation of MAO-A was found to be statistically significant at 100, 200, 400, and 800 *μ*M arecoline treatments compared with the control group (0 *μ*M) (*P* < 0.05); the change in downregulation was particularly significant at 800 *μ*M arecoline in the Ca9-22 cancer cell line (the mean fold change ± standard errors (SE) was −8.55 ± 0.33). When the arecoline dose was gradually increasing, a decreasing trend effect (*P* < 0.0001) for MAO-A expression could be observed.

### 3.3. The MAO-A and MAO-B mRNA and Protein Expression of Paired Tissue in Oral Cancer Patients

**Figure 3:**
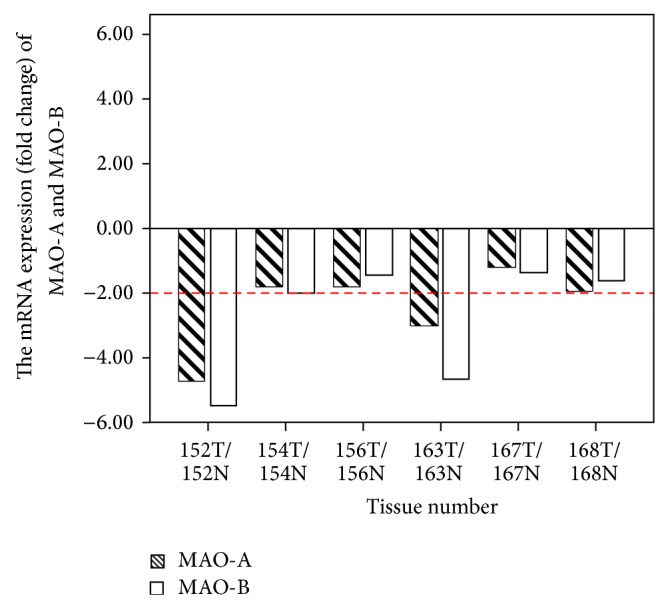
The mRNA expression of MAO-A and MAO-B in human oral tumor (T) tissues compared with their adjacent normal (N) tissues. The relative fold change was estimated by the formula 2^−ΔΔ*C*_*p*_^ compared with adjacent tissue (*N* = 6).

**Figure 4:**
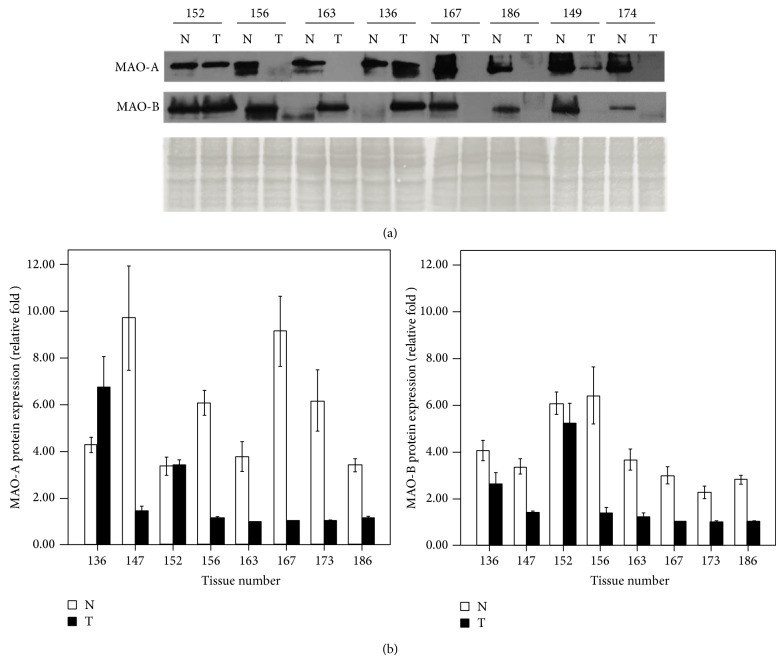
(a) The induced protein expression of MAO-A and MAO-B of human oral tumor (T) tissues compared with their adjacent normal (N) tissues. (b) The protein expression of MAO-A and MAO-B was presented by relative fold; the average fold (mean ± SE) was calculated in triplicate.

In comparison with their adjacent noncancerous tissues, the downregulation mRNA of MAO-A and MAO-B for cancer tissues were observed in patients numbers 152, 154, 156, 163, 167, and 168 (Figure 3). Using Western blotting, we investigated MAO-A and MAO-B quantitative protein expression from eight patients (numbers 136, 149, 152, 156, 163, 167, 174, and 186) (Figure 4). Compared with their adjacent noncancerous tissue, downregulation of protein expression of MAO-A and MAO-B in cancerous tissue was shown in patients numbers 149, 156, 163, 167, 174, and 186, excluding number 136 and number 152. MAO-A expression was higher in number 136 cancer tissue than in its adjacent tissue, but MAO-B expression was lower in cancer tissue than in the adjacent tissue. In number 152 cancer tissue, slightly increased expression of MAO-A and decreased expression of MAO-B were found.

### 3.4. MAO-A Single Nucleotide Polymorphism (SNP) Analysis

We selected a total of 260 males with oral and pharyngeal cancer and 68 males with OPMDs. All of these participants have a habit of BQ chewing.  Table 1 shows that compared with OPMDs patients, BQ chewers who had the MAO-A A-allele (SNP rs2283725) had an increased risk of oral cancer (OR = 1.69; 95% CI = 0.98–2.90), although at borderline significant level. BQ chewers who had the MAO-A G-allele (SNP rs5953210) had a significantly increased risk (OR = 1.76; 95% CI = 1.02–3.01) of oral cancer (*P* < 0.05).

## 4. Discussion

BQ chewing is an emerging health-associated issue in Asia and the South Pacific islands, as well as among diverse migrant populations in western countries. This is the first study to indicate that variants of the MAO gene may be related to BQ-related oral and pharyngeal cancer occurrence. The MAO gene is present in human blood and neuron synapses, and it catalyzes deamination effects of biogenic amines and regulates the concentration of several neurotransmitters (such as dopamine, serotonin, norepinephrine, and catecholamines) in the central nervous system, which plays an important role in physiology and behavior [19]. The MAO gene is divided into two types. The MAO-A gene is primarily responsible for the metabolism of serotonin and norepinephrine and may indirectly affect mood and impulse control; the MAO-A gene is a major determinant of MAO activity [20]. The MAO-B gene is associated with the metabolism of dopamine and phenylethylamine [20].

Past studies only focused on the association of the MAO-A gene with other cancers and never explored its association with oral pharyngeal cancer, specifically. Also, the relationship between MAO-B and cancer has rarely been mentioned. Mikula et al. found that downregulation of MAO-A may be associated with the occurrence of colon cancer [21]. MAO-A gene was downregulated in lymph node status (*N*_0_) of gastric cancer [22]. In prostate cancer, Peehl et al. noted that there is a high expression of MAO-A in patients with a high tumor grade [23]; the targeting of antidepression drugs on MAO-A may provide potential future applications in the treatment of prostate cancer [24]. Two studies have shown that MAO-A gene expression is suppressed in cholangiocarcinoma patients [25, 26]. A case-control study also found that tumor cells had high concentrations of metanephrine in patients with pheochromocytoma, which may be due to the downregulation of MAO-A [27]. Rybaczyk et al. found that MAO-A exhibited significantly lower expression in cancerous tissue than its noncancerous control tissue in human, mouse, and zebrafish studies [28].

To our knowledge, population data regarding the relationship between the MAO gene and oral and pharyngeal cancer among BQ users has not been available. Previous studies have indicated that arecoline can cause many adverse effects in cells, such as cytotoxicity, carcinogenicity, immunotoxicity, and genotoxicity [8–11]. Arecoline may be metabolized by the MAO gene* via* xenobiotic metabolism, which is involved in phase I biotransformation. To simulate the oral cells of Taiwanese men, we cultured normal HGF cells from the biopsy specimens. In the* in vitro* model, our results suggested that in primary cell culture of HGF, treatment with arecoline may increase expression of MAO-A in a dose-dependent manner. Conversely, Ca9-22 cancer cells treated with higher concentrations of arecoline may induce downregulation of MAO-A.

**Figure 5:**
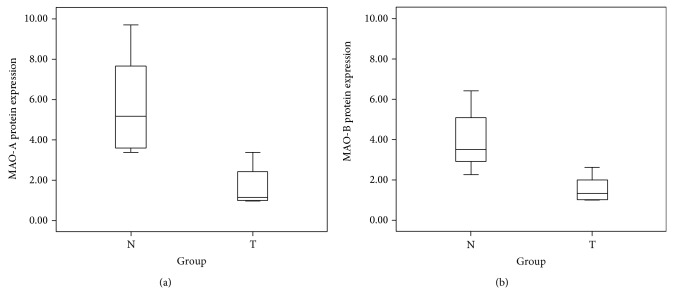
Boxplot of protein expression among 8 patients. (a) MAO-A protein expression between oral tumor (T) tissue and the oral normal (N) adjacent tissue; (b) MAO-B expression between oral tumor (T) tissue and the oral normal (N) adjacent tissue.

To exclude individual differences of gene expression in humans, paired tissues from oral cancer patients were used to explore the mRNA and protein expression of MAO-A and MAO-B. The clinical characteristics and substance use status (alcohol, betel, and cigarette use) of the oral and pharyngeal patients were shown in Table 2. Overall, in the* in vivo* model, our data indicated that patients (numbers 152, 154, 156, 163, 167, and 168) showed consistent downregulation of MAO-A and MAO-B mRNA in oral cancer tissue compared with noncancerous adjacent tissue. Decreased MAO-A and MAO-B protein expression was found to be statistically significant in cancerous tissue compared with adjacent noncancerous tissue among 8 patients using the nonparametric Wilcoxon signed-rank test (*P* < 0.05, Figure 5).

Generally, the protein expression of the MAO gene exhibited downregulation in oral cancer tissue compared with noncancerous tissue, and this performance is consistent with mRNA levels. This pattern was also observed in the cell model; the normal HFG cells with higher doses of arecoline showed higher mRNA expression, and the cancerous Ca9-22 cells with higher doses of arecoline showed lower mRNA expression. We speculated that the differences of mRNA expression observed between normal HFG cell and cancer Ca9-22 cell may be associated with the cancer type, but this phenomenon needs to be confirmed in further study.

The MAO-A gene is located on chromosome Xp11.3, and the MAO-B gene is located on the chromosome Xp11.23 region. In this study, one SNP (rs2283725, located on intron 3) of MAO-A was selected to analysis. The other one located on the 5′ intergenic region (rs5953210, located on 5′ near gene) was also included to allow ascertainment of linkage disequilibrium (LD) extent beyond the gene boundaries. Previous reports have indicated OPMDs are significant predictors for malignant transformation to oral and pharyngeal cancer [5, 29, 30]. BQ chewers with both MAO-A SNPs, rs2283725 and rs5953210, were associated with the risk of oral and pharyngeal cancer occurrence compared with OPMDs. The rs5953210 risk G-allele was significantly associated with the risk of oral and pharyngeal cancer (OR = 1.76; 95% CI = 1.02–3.01) and the rs2283725 risk A-allele at borderline significant level (OR = 1.69; 95% CI = 0.98–2.90). Overall, our results suggest that MAO-A variants may contribute to genetic susceptibility to oral and pharyngeal cancer in BQ chewers. In addition, BQ chewers with risk allele combined with cigarette or alcohol use significantly increased the risk of oral and pharyngeal cancer (data not shown). A case-control study of 2,572 Caucasian men suggested that a rare 5-copy variation of the MAO-A variable-number tandem repeat (VNTR) genotype may be associated with the development of prostate cancer; the frequency of the rare 5-copy variation in the case group (0.5%) was lower than the frequency in the control group (1.8%) and reduced the risk of prostate cancer (OR = 0.30; 95% CI = 0.13–0.71) [31]. A previous report indicated that, in gastric cancer, changes to the MAO-A gene may be related to the DNA copy number of cancerous tissue with a statistically significant linear correlation [32]. Limitations of this study were small sample size to present the expression of MAO-A mRNA and protein. A large size of sample was needed to confirm expression of MAO-A and MAO-B in betel quid-related oral and pharyngeal cancer. Additionally, we cannot take OPMDs specimen for further research, because it is very difficult to recruit OPMDs patients willing to undergo surgery and sign the informed consent.

In conclusion, this report is the first study to consider how downregulation of the MAO gene family (MAO-A and MAO-B) and MAO-A SNP variants play an important role in the occurrence or development mechanism of oral and pharyngeal cancer. Our previous report demonstrated that BQ chewing may significantly produce ROS, which may contribute to oxidative injury of oral tissue [33]. A screen tool of MAO at-risk variations may be useful to prevent the occurrence of oral and pharyngeal cancer among BQ chewers. In the future, these studies may provide new insight into the relationship between malignant transformation of OPMDs and oral and pharyngeal cancer in the modulation of MAO gene activity associated with ROS.

## Figures and Tables

**Table 1:** Distribution of BQ chewers with MAO-A allele types between oral and pharyngeal cancer and OPMDs male patients.

	Oral and pharyngeal cancer		OPMDs		P^a^	OR (95% CI)
	*N*	(%)^a^	*N*	(%)		
BQ chewers						
rs2283725						
Allele						
G	95	(36.5)	33	(49.3)	0.0572	1.00
A	165	(63.5)	34	(50.7)		1.69 (0.98–2.90)
rs5953210						
Allele						
A	94	(36.3)	34	(50.0)	0.0393	1.00
G	165	(63.7)	34	(50.0)		1.76 (1.02–3.01)^b∗^

^a^Statistical *P* values were estimated by chi-square (*χ*²) test; ^b∗^ *P* < 0.05.

BQ: betel quid; OPMDs: oral potentially malignant disorders; OR: odds ratio; 95% CI: 95% confidence interval.

**Table 2:** Clinical characteristics and substance use status comparison of male BQ chewers with oral cancer (*N* = 10).

Number	Age	Tumor site	ICD 9 code	TNM	Stage	Pathological diagnosis	A^a^	B^b^	C^c^
136	45	Tongue	141	T2N1M0	III	SCC^d^	+	+	+
149	71	Oral	145.9	T2N0M0	II	Verrucous carcinoma	−	+	+
152	46	Tongue	141	T2N0M0	II	SCC	+	+	+
154	39	Buccal	145	T3N0M0	III	SCC	+	+	+
156	57	Buccal	145	T3N0M0	III	SCC, grand II	NA	+	+
163	39	Oral	145.9	T2N0M0	II	SCC, grand II	−	+	+
167	45	Tongue	141	T2N0M0	II	SCC, grand II	+	+	+
168	56	Buccal	145	T2N0M0	II	SCC	+	+	−
174	45	Buccal	145	T4N1M0	IV A	SCC	−	+	+
186	46	Buccal	145	T4N1M0	IV A	SCC	+	+	+

^a^Alcohol use.

^b^Betel use.

^c^Cigarette use.

^d^SCC: squamous cell carcinoma.

NA: no information can be available.

## Abbreviations

BQ: Betel quid
AN: Areca nut
OPMDs: Oral potentially malignant disorders
MAO: Monoamine oxidase
SNP: Single nucleotide polymorphism.
